# Informed Consent for Braces

**DOI:** 10.5005/jp-journals-10005-1246

**Published:** 2014-08-29

**Authors:** Vikas Jharwal, Mridula Trehan, Nidhi Rathore, Pooja Rathee, Deepesh Agarwal, Nikunj Mathur

**Affiliations:** Senior Lecturer, Department of Orthodontics and Dentofacial Orthopedics Mahatma Gandhi Dental College and Hospital, Jaipur Rajasthan, India; Professor and Head, Department of Orthodontics and Dentofacial Orthopedics Mahatma Gandhi Dental College and Hospital, Jaipur Rajasthan, India; Senior Lecturer, Department of Orthodontics and Dentofacial Orthopedics Mahatma Gandhi Dental College and Hospital, Jaipur Rajasthan, India; Assistant Professor, Department of Orthodontics and Dentofacial Orthopedics Government Dental College, Jaipur, Rajasthan, India; Reader, Department of Orthodontics and Dentofacial Orthopedics Jaipur Dental College, Jaipur, Rajasthan, India; Assistant Professor, Department of Oral Medicine and Radiology, Government Dental College, Jaipur, Rajasthan, India

**Keywords:** Consent, Informed consent, Ethics, Medicolegal perspective

## Abstract

The influence of law on the orthodontic profession has greatly increased in the last few decades. Dental law has emerged today as a full-fedged specialty dealing with a variety of areas, like professional negligence, doctor-patient contracts, consumer protection laws, ethics, general and special health legislations and practice regulatory mechanisms. This article highlights the concept of informed consent which is based on the premise that each individual has a right to make decisions concerning his health, disease and treatment.

**How to cite this article:** Jharwal V, Trehan M, Rathore N, Rathee P, Agarwal D, Mathur N. Informed Consent for Braces. Int J Clin Pediatr Dent 2014;7(2):105-108.

## INTRODUCTION

The Webster’s dictionary defines consent as ‘to give assent or approval’. The over simplification of Webster’s definition however does not and should not apply to the field of dentistry.^1^ The British Dental Association (BDA) ‘Ethics in Dentistry’ advice sheet is a case in point. The concept of medical informed consent is evident already in the Hippocratic Oath, which clearly illustrates the notion that respect is an integral part of the relationship between patients and healthcare professionals in the pledge, ‘first, do no harm’.^2^

## CONSENT TO DENTISTRY

Informed consent is not a signature on a consent form. It is not a single event, it is a process of dialog between the dentist and the patient. The British Dental Association ‘Ethics in Dentistry’ advice sheet defines the process of expressing consent as ‘a patient gives express consent when he or she indicates orally or in writing consent to undergo examination or treatment or for personal information to be processed’.^2,3^

## TYPES OF CONSENT

The BDA Ethics in Dentistry further lays down the following definitions:^2,3^

- *Implied consent:* Where the patient indicates agreement to examine by lying in the dental chair and opening the mouth.
- *Valid consent:* For consent to be valid, it must be specific, informed and normally be given by a patient, or a parent or a guardian.
- *Written consent:* It is to be taken for major procedures, examples being in orthodontics, therapeutic extraction, orthognathic surgery, orthodontic mini-implant placement, sedation, etc. Written consent is all the above with a signature of the patient. Written consent is important but cannot be considered a substitute for obtaining valid consent. This brings us to the obvious question of the right age of patient for giving consent.
- *Informed consent:* Requires a full explanation of the nature, purpose and material risks of the proposed procedures in a language that the patient understands. The patient should have the opportunity to consider the information and ask questions in order to arrive at a balanced judgment of whether to proceed with the proposed treatment.

The following information is provided to patients who will be starting orthodontic treatment in dental office: While recognizing the benefits of teeth that function well and have a pleasing appearance, a patient should be aware that orthodontic treatment, like any other treatment of the human body, has inherent risks and limitations. If a patient decides not to proceed with treatment, then the state of the dentition (teeth) can be expected to continue on its present path. But, the rate of these changes is very unpredictable. A patient must balance the risks of nontreatment against the risks of treatment. In our opinion, the risks of treatment are not enough to rule against proceeding; nevertheless, a patient should consider them carefully before they make a final decision.^4,6^

## PATIENT’S RESPONSIBILITIES

Orthodontic treatment will not be completely successful unless a patient complies with the directions given by us. Many treatment forces are applied by our patient outside of the office. A patient needs to fulfill their responsibilities because the patient’s effort is equal to their treatment result. It is just that simple! Typically, these responsibilities will include the following:

- * Correct use of the appliance:* Orthodontic appliances are designed to deliver forces in a very specific manner. If the appliance is not worn as requested, the treatment will not proceed as planned.
- * Meticulous oral hygiene:* A thorough brushing several times each day, complete fossing once each day and daily application of a fluoride mouth rinse are important.
- * Care of the appliance:* Lost, broken or bent appliances disrupt the treatment plan. Unwanted tooth movement may occur, if the appliance is not working as designed.
- * Regularly scheduled appointments:* Appliances must be adjusted periodically and treatment progress must be monitored carefully. Missed or rescheduled appointments prolong treatment inevitably. Some appointments will be during work or school hours.
- * Routine dentalvisits:* An orthodontic patient should continue to consult his/her family dentist for regular cavity check-ups, teeth cleaning, sealants and fluoride varnish applications and periodontal evaluations based on their risk assessment.^4,6,9^

## TOOTH DECAY/STAINS/DECALCIFICATION

The bacteria present in plaque (the white, sticky material that is constantly forming on tooth surfaces) release acids that draws the calcium and phosphorous out of the outer surface of the tooth. This will damage patient’s tooth surfaces if the plaque is not removed several times each day by thorough brushing, fossing and rinsing. This damage includes tooth decay and permanent white decalcification markings. The bacteria that live in plaque thrive on refined carbohydrates (sugar!). While a patient is undergoing orthodontic treatment, they should minimize the amount and frequency of sugar in their diet. These same problems can occur without orthodontic treatment, but the risk is greater to an individual wearing braces.^4,6,9^

## INFLAMMATION OF THE SOFT TISSUES

The wires, brackets or band attachments can sometimes irritate the lips, cheeks or gum tissue. These soft tissue irritations usually heal quickly. The wax that we give our patients can help cushion these irritations while they are healing. If the irritation is persistent, a patient should contact us immediately so that we can solve the problem. Poor oral hygiene during orthodontic tooth movement can accelerate deterioration of the periodontal tissues (gums and underlying bone). Severe tissue reactions may require us to refer a patient to a periodontal (gum) specialist or discontinue orthodontic treatment.^4,6,9^

## TREATMENT GOALS AND TREATMENT COMPLETION

We have tried to establish realistic and achievable goals for treatment. We know that patients share our desire to produce the best result that is possible. As we begin treatment, we believe that we will be able to achieve those goals. Nevertheless, unforeseen factors, such as atypical tooth formation and disproportionate jaw growth may interfere with our intentions. These biological processes are beyond the orthodontist’s control. As treatment proceeds, we will keep a patient fully informed as to treatment progress. If our original goals become unreachable, we will discuss the alternatives with the patient.^4,6,9^

## TREATMENT DURATION

Although we give an estimate to a patient of his/her treatment time, we do not know exactly how long his/her treatment will take. Individuals vary considerably in their response to orthodontic forces, so treatment time may be more or less than our estimate. It is our general intention to have the treatment move along in a fashion that is consistent with tissue health, minimal discomfort and long-term stability.^6,10^

## PAIN OR DISCOMFORT IN THE JOINT OF THE JAW

Pain, discomfort, clicking or popping noises may occur in or near the joint of the jaw at any time, including during orthodontic treatment. Just as with any joint discomfort, the possible causes vary widely. Orthodontic treatment did not provide risk to the development of signs and symptoms of TMD, regardless of the technique used for treatment, the extraction or nonextraction of premolars and the type of malocclusion previously presented by the patient. It is important that we be told about jaw joint problems so that we can deal with them promptly.^6,9,10^

## LOSS OF TOOTH VITALITY

It is possible for the nerve of a tooth to die during orthodontic treatment, especially if a tooth was previously injured, bumped or impacted. It is helpful in our monitoring the health of each tooth for a patient to tell us about any previous injury or stress to the tooth. Sometimes seemingly minor bumps can result in nerve damage that is unknown to a patient. Such previous injuries cannot always be detected during the orthodontic diagnostic process. Root canal treatment may be recommended if such a problem occurs. Extraction is necessary occasionally, though not usually.^6,10^

## INJURY FROM APPLIANCES

Appliances are designed to have a maximum amount of strength and a minimum amount of injury potential. Nevertheless, accidents can occur and a patient can be injured by sharp parts of the appliances. A patient could also be injured during a routine appointment by one of our sharp instruments. It is also possible for a patient to swallow or inhale small parts of the appliance that fall into the back of the throat at any time, including routine office visits. Everyone working with a patient in the office will be attentive particularly to preventing accidents. Headgear instructions must be followed carefully. Improperly handled headgear may cause injury to the face or eyes, even blindness. Patients are warned not to wear the headgear during times of horseplay or competitive activity. Always release the elastic force before removing the headgear from the teeth. Although our headgear is equipped with a safety system, we urge caution at all times. Tender teeth should be expected after in office adjustments. The period of tenderness or sensitivity varies with each patient and the procedure performed. Typically, post adjustment tenderness may last 24 to 48 hours. Abnormal wear of tooth structures is also possible if a patient grinds his/her teeth excessively.^5,6,10^

## UNEXPECTED GROWTH CHANGES

Growth of the facial structures and the teeth can sometimes take unexpected turns. A child who has grown in average proportions may not continue to do so. If growth becomes disproportionate, the jaw relationships can be affected. If this occurs, original treatment objectives may not be met.^6,10^

## UNEXPECTED TOOTH ERUPTION PROBLEMS

Sometimes when a tooth is erupting (growing in), it does not follow the usual and expected direction of eruption. The tooth may not be able to reach its normal position and will become impacted or stuck under the bone. Usually, it is possible to solve these impactions, but not always. If the impaction is extreme and the tooth becomes tightly bound to its surrounding bone, it may not be possible to move that tooth at all. We will be monitoring the teeth carefully as they grow into catch such a problem. If a tooth does become impacted, a change in the plan of treatment may be necessary.^6,10^

## ENAMEL FRACTURES

Tooth enamel is a crystalline structure and like other crystals, it can have undetected defects and fracture lines within it. As a result, even when extreme care is taken, enamel can fracture during placement or removal of the appliances. Such fractures may also occur if a band or bracket is bitten on at just the wrong angle or if the enamel has been weakened by decay or dental restorations (fillings). The enamel may also be damaged by rubbing against a part of the appliance. Tooth colored ceramic brackets are abrasive to enamel. A patient must be sure to report if any tooth is bearing against a ceramic bracket.^6,10^

## ORAL SURGERY

Sometimes tooth removal or orthognathic (jaw) surgery is necessary in conjunction with orthodontic treatment, especially to correct crowding or severe jaw imbalances. We will recommend these procedures only if it improves the prospects for successful treatment. Risks involved with treatment and anesthesia should be discussed with the patient’s general dentist or oral surgeon before making a decision to proceed with this procedure.^6,10^

## SUCCESS OF THE TREATMENT

We intend to do everything possible to provide the very best treatment result. However, we cannot guarantee that the proposed treatment will be successful to a patient’s complete satisfaction. Individual patient differences create the possibility of incomplete or unstable results. Selective retreatment may be necessary despite the very best of care.^6,10^ Emphasis must be placed on early, timely intervention, parental involvement, effective communication, cultural competence, and the ‘medical/dental home’ concept as methods to reduce negative dental attitudes and behaviors of children.^11^ Although some patients were happy with the way, their consent was obtained examples were also given of lack of information, confusion and even of deceit.^12^

## ADDITIONAL TREATMENT

Unforeseen circumstances, such as growth changes or gum disease, may cause us to recommend a form of treatment not previously discussed. These changes in treatment plan may require additional treatment in our office or with another specialist. Additional treatment with associated fees will be discussed with the patient.^7,6,10^

## ROOT RESORPTION

Orthodontic forces initiate a cellular response in the supporting tissues surrounding the roots of the teeth. It is this cellular response that allows the teeth to move. Sometimes, this response becomes confused resulting in damage to the ends of the roots of the teeth. Usually, this effect is mild and does not compromise the teeth. However, sometimes this root resorption can be extensive and may then endanger the teeth, if periodontal (bone and gum) support is lost at some future time. Some patients are prone to this happening, some are not. Because it is not possible to predict which teeth might be affected, we may want to take progress X-rays of a patient’s teeth during the treatment process to evaluate whether root resorption is occurring.^6,10^

## STABILITY OF THE FINAL RESULT

The tooth positions achieved at the end of treatment may not be perfectly stable. The retainers that, a patient wears, will enhance the stability of the final result, but even diligent wear of the retainers may not keep a patient’s teeth exactly as they were at treatment’s end. The teeth and jaw structures are dynamic system that constantly changes throughout one’s lifetime. Orthodontic treatment does not make a patient immune to this process. Maturational changes that occur after active orthodontic treatment may alter the quality of the end result. Ongoing wear of the retainers will minimize changes. If a patient decides to stop wearing, his/her retainers at some point, his/her teeth may change. Some of the original problem may re-emerge.^6,8,10^

## CONCLUSION

Clearly, there are a number of sources of potential iatrogenic damage to the patient during orthodontic treatment. However, severe damage is rare. Severe malocclusions have more to benefit from treatment than less severe malocclusions, and motivation between such groups may vary. Individuals should be assessed for risk factors for all aspects of care content and recommendations are analyzed and discussed from a medicolegal perspective. It is important that such guidelines are updated in the light of research findings and clinical audit. Clinicians should be aware of such guidelines and the legal implications of failing to at least consider when providing advice and/or treatment for patients.
